# Emerging Perspectives in Scaffold for Tissue Engineering in Oral Surgery

**DOI:** 10.1155/2017/4585401

**Published:** 2017-02-26

**Authors:** Gabriele Ceccarelli, Rossella Presta, Laura Benedetti, Maria Gabriella Cusella De Angelis, Saturnino Marco Lupi, Ruggero Rodriguez y Baena

**Affiliations:** ^1^Department of Public Health, Experimental Medicine and Forensic, Human Anatomy Unit, University of Pavia, Via Forlanini 8, 27100 Pavia, Italy; ^2^Centre for Health Technologies (CHT), University of Pavia, Via Ferrata 5, 27100 Pavia, Italy; ^3^Department of Clinico-Surgical, Diagnostic and Pediatric Sciences, School of Dentistry, University of Pavia, P.le Golgi 2, 27100 Pavia, Italy

## Abstract

Bone regeneration is currently one of the most important and challenging tissue engineering approaches in regenerative medicine. Bone regeneration is a promising approach in dentistry and is considered an ideal clinical strategy in treating diseases, injuries, and defects of the maxillofacial region. Advances in tissue engineering have resulted in the development of innovative scaffold designs, complemented by the progress made in cell-based therapies. In vitro bone regeneration can be achieved by the combination of stem cells, scaffolds, and bioactive factors. The biomimetic approach to create an ideal bone substitute provides strategies for developing combined scaffolds composed of adult stem cells with mesenchymal phenotype and different organic biomaterials (such as collagen and hyaluronic acid derivatives) or inorganic biomaterials such as manufactured polymers (polyglycolic acid (PGA), polylactic acid (PLA), and polycaprolactone). This review focuses on different biomaterials currently used in dentistry as scaffolds for bone regeneration in treating bone defects or in surgical techniques, such as sinus lift, horizontal and vertical bone grafts, or socket preservation. Our review would be of particular interest to medical and surgical researchers at the interface of cell biology, materials science, and tissue engineering, as well as industry-related manufacturers and researchers in healthcare, prosthetics, and 3D printing, too.

## 1. Introduction

Bone tissue engineering aims to restore tissues damaged due to trauma, diseases, or congenital abnormalities. This tissue engineering approach can be developed by combining stem cells with innovative scaffolds designed to produce the required extracellular matrix in an adequate manner and ultimately a healthy bone tissue with acceptable geometry, size, and composition [1–3]. Thus, regenerative medicine can be successfully combined with tissue engineering to recreate the appropriate cellular microenvironment that can rebuild whole organs [4, 5].

In the last 20 years, there has been increasing interest in tissue regeneration in the craniofacial region as well as for whole teeth and periodontal structures in dentistry [6]. The principles of tissue engineering have found widespread application in several branches of dentistry, such as periodontics, oral maxillofacial surgery, and implant dentistry [2, 7]. Tooth engineering attempts to enhance the creation of osseointegrated implants with specific, biocompatible materials that replace the missing tooth or provide support to the regenerated maxillary bone. In implant dentistry, the lack of adequate bone tissue and proximity to important anatomical structures (such as the maxillary sinus and the inferior alveolar nerve) are the most frequently observed problems at the implantation site [7].

To avoid iatrogenic damage to sensitive structures, the sinus lift, horizontal and vertical bone grafts, and socket preservation are the conventionally used surgical techniques [8, 9]. These techniques have been considerably improved in the last 5 years to achieve the regeneration of increasingly larger and higher bone volume [10, 11] and will ensure the best prosthetic rehabilitation for each patient. For successful outcomes in regenerative dentistry, the combination of stem cells of mesenchymal origin and scaffolds has been regarded to have considerable potential in regenerative medicine at the maxillary bone level [12–14].

For these reasons, bone tissue engineering represents an important and promising approach to treat various pathological conditions in the oral cavity. Bone tissue engineering for pathological bone conditions requires an appropriate source of mesenchymal stem cells, such as dental pulp stem cells (DPSCs) or periosteal stem cells (PSCs), and a suitable scaffold on which the stem cells can be seeded and growth factors/molecular signals can be provided in order to facilitate bone regeneration [1–5, 15]. Several studies have reported different scaffolds for different types of tissue regeneration; for example, some scaffolds used in plastic surgery attract the epidermal and connective substitutes, while others are specifically used for bone regeneration [16, 17].

This review will focus on the significant advancements that have been made in the field of dentistry-based tissue engineering with emphasis on the different biomaterials currently available in clinical therapy for surgical procedures in the oral cavity. Moreover, it would be of particular interest to medical and surgical researchers at the interface of cell biology, materials science, and tissue engineering, as well as industry-related manufacturers and researchers in healthcare, prosthetics, and 3D printing, too.

## 2. Stem Cells in Dentistry-Based Tissue Engineering

Stem cells can be classified as totipotent, pluripotent, or multipotent according to their abilities to differentiate into other cell types [2, 6, 49]. Totipotent cells can give rise to the whole organism; pluripotent cells (iPSC, such as embryonic stem (ES) cells) can give rise to every cell type of an organism except its extraembryonic tissues, such as the placenta. Multipotent stem cells (MSC) are adult stem cells, which can differentiate into a specific cell lineage [50]. Owing to ethical reasons and technical issues associated with ES cells and iPSC, MSCs are the most commonly used stem cells in tissue engineering, including dentistry-based tissue engineering [49]. MSCs can be isolated from several tissues, such as bone marrow, peripheral blood, umbilical cord blood, adult connective tissue, dental tissues, placenta, and amniotic membrane [1, 2, 50, 51]. Morphologically, MSCs adhere to plastic, have fibroblast-like appearance, and are able to differentiate into osteocytes, chondrocytes, and adipocytes [52, 53]. MSCs express surface-specific antigens, such as CD105, CD73, and CD90, and they are negative for the leukocyte marker (CD45) and the primitive hematopoietic progenitor and endothelial cell marker, CD34 [54]. Several types of adult stem cells with mesenchymal origin have been isolated from the oral cavity, including DPSCs [55, 56], stem cells from human exfoliated deciduous teeth (SHED) [57], periodontal ligament stem cells (PDLSCs) [58], dental follicle progenitor stem cells (DFPCs) [6], and stem cells from apical papilla (SCAP) [6, 59].

Gronthos et al. first identified DPSCs with a high cell proliferation capacity and the ability to differentiate into osteoblasts and chondroblasts [60]. In addition, when transplanted in host mice, DPSCs can differentiate into odontoblast-like cells and form some important tooth structures, such as dentin-like structure or cementum, when cultured on ceramic substrates (such as hydroxyapatite or tricalcium phosphate) [61]. Several in vivo applications of DPSCs have improved bone regeneration, as demonstrated by D'Aquino et al. based on radiographic evidence [56] and by Graziano et al. based on histology [62]. Moreover, DPSCs seeded onto collagen scaffolds in the presence of dentin matrix protein 1 formed an organized matrix [61, 62], which could induce the formation of hard tissue.

Exfoliated deciduous teeth are another important source for stem cells, from which Miura et al. isolated stem cells with mesenchymal properties [63]. Similar to DPSCs, SHED express MSC markers, including CD105, CD146, Stro-1, and CD29 and when transplanted, they form a dentin-like structure [63]. SCAP (from apical papilla) were isolated from the root apex of a developing tooth and showed MSC properties, such as the expression of surface antigen markers (they also express CD24); SCAP can undergo differentiation to form odontoblasts and chondroblasts [64]. PDLSCs, isolated from the dental follicle during tooth development, are highly positive for mesenchymal markers; they can differentiate into osteoblasts, chondrocytes, adipocytes, neurons, and even hepatocytes [6, 58].

Finally, the periosteum from craniofacial bones has also been proposed to be a source of progenitor cells responsible for injury repair in adult bones. In fact, periosteal cells (PCs), described by Mattioli-Belmonte et al. as cells with MSC-like properties, are involved in cell mechanosensing and contribute to matrix organization, bone microarchitecture, and bone strength [65, 66]. In addition, it has been demonstrated that PCs could replace mesenchymal cells from bone marrow in oral tissue engineering applications owing to the ease of collecting and the rapid in situ engraftment of PCs [66]. It has been proposed to culture PCs ex vivo and subsequently seed them into a natural or synthetic scaffold [67]. However, the success of this approach is strictly limited to the use of an appropriate material that is able to enhance the differentiation of PCs.

Thus, in dentistry-based bone tissue engineering, scaffolds in combination with the appropriate stem cell are considered to be the most important factors to create substitutes for the original tissue after any injury.

## 3. Scaffolds

### 3.1. Classification of Scaffolds

In scientific literature, the term “scaffold” has been adopted to indicate a biomaterial that can provide support. Here, “support” is used to describe the biomaterial as a biological platform that facilitates the appropriate repair and restoration of the physiological/histological features of injured tissues during the healing process [16, 17, 23, 62].

In tissue regeneration, a biocompatible scaffold will allow cell adhesion and induce cell proliferation and differentiation without triggering any inflammatory responses or rejection from the body [23]. Currently, the ultimate goal of tissue engineering is to create a three-dimensional (3D) biocompatible support that can be inserted into a tissue to repair a lesion or correct a defect by allowing the adhesion and proliferation of a specific cell type [23].

An ideal bone scaffold must have three fundamental features: it should be osteogenic, osteoconductive, and osteoinductive. An osteogenic material can generate bone tissue, which is a characteristic unique to osteoblasts. Thus, the “living” bone can be considered the only real osteogenic scaffold. Moreover, to ensure that the osteogenic feature is retained in bone grafts, the graft must be collected and used as quickly as possible to facilitate cell survival after surgical trauma [17]. An ideal scaffold should also be an osteoconductive material that stimulates bone cells to grow on its surface. Moreover, the osteoinductive capacity (the ability of the biomaterial to induce proliferation and differentiation of MSCs in preosteoblasts, which produce matrix) is essential for bone healing.

These fundamental characteristics alone are insufficient for the successful in vivo application of the bone scaffold. To fabricate a bone scaffold, an ideal biomaterial must also possess other properties, such as being bioinert, biocompatible, bioactive, and biodegradable with suitable mechanical properties. Furthermore, the biomaterial should also be able to withstand sterilization to prevent infections and be interconnected and demonstrate controlled porosity [17]. Moreover, it should be able to undergo efficient resorption at the same time that the bone regenerates.

The scaffold-cell interaction must also ensure easy penetration, distribution, and proliferation. The biomaterial should be 90% porous with a suitable pore diameter to enable the cells to penetrate the biomaterial, thus, ensuring the growth of new bone tissue and optimal vascularization [17]. Biocompatibility, however, remains the most essential property that scaffolds must possess; in fact, the biomaterials should not induce inflammatory responses in the body or show any immunogenicity or cytotoxicity [24]. Furthermore, from the industrial point of view, the manufacturing process should be simple, fast, and cost-effective. However, it has been considered difficult and challenging to fabricate a single ideal biomaterial that encompasses all these features.

Finally, it is also essential that the scaffold biomaterial must be efficiently resorbed with the deposition of new bone tissue so that the new bone can replace it entirely, while maintaining the shape and thickness [68]. Craniofacial scaffolds (having several applications in dentistry) must fill three-dimensionally complex defects and provide adequate resistance to temporary load during regeneration. To meet these requirements, it is fundamental to apply the modulus of elasticity (or Young's modulus “E”), described as the ratio between the stress applied and the resulting deformation in the biomaterial [18, 69, 70]. Since skull bones have an elastic modulus between 100 and 30000 MPa (which can be variable in relation to the bone type and load zone), craniofacial scaffolds must have a similar elastic modulus to ensure resistance to the load without breaking [68]. The modulus of elasticity of the material increases with its volume fraction, but increased volume can decrease the permeability and, consequently, the porosity of the material. Then, the volume (and density) of the material will be directly proportional to its elasticity and inversely proportional to its permeability. This indicates that it is difficult to provide good elasticity and resistance to the biomaterial, while concomitantly maintaining an optimal permeability for cell colonization [19].

The numerous and different types of scaffolds have been predominantly classified according to the intrinsic characteristics of the biomaterials and placed in macrogroups [20]. For simplicity, we will discuss bone grafts, matrices, polymeric materials, and combined (composite) scaffolds.

### 3.2. Bone Grafts

Bone grafts are classified as autologous, homologous, heterologous (xenografts), and alloplastic grafts. Currently, autologous bone graft is considered as the gold standard among all bone grafts. In autologous bone grafting, the donor's own bone is taken from a healthy part of the donor's body and grafted in the affected region, minimizing rejection issues. The donor sites can be intraoral or extraoral [21, 70]. It is interesting to note the different properties of the cortical and cancellous bone grafts. A cortical bone graft provides good structural support and reduced resorption. Nevertheless, due to its high density, revascularization of the newly formed tissue is slow and difficult resulting in engraftment delay. In contrast, a cancellous bone graft ensures early revascularization, resulting in faster engraftment, lesser risk of infection, and a shorter time for implant placement.

The allografts or allogeneic grafts are derived from donors of the same species (usually from bone banks) but lose many of their characteristics during the long and expensive process of sterilization and decellularization. Although these processes are necessary to minimize rejection or disease transmission, they deprive the bone of its osteogenic properties, making it a simple, empty scaffold. These grafts can be further classified as freeze-dried bone allograft (FDBA) and demineralized freeze-dried bone allograft (DFDBA) [22]. Due to their high manufacturing costs and increased rate of resorption these types of grafts are limited to small and medium defects used [25].

Xenografts are a cheaper alternative to allografts but undergo similar sterilization procedures. They are animal-derived grafts, mainly obtained from cattle, pigs, and horses. Various studies have shown that these materials provide support and survival similar to those of autologous bone grafts, but without the osteogenic properties. The deproteinized bovine bone in sterilized granules or blocks is an example of a xenograft commonly used in bone regeneration [26, 27].

Several alloplastic grafts, such as *β*-tricalcium phosphate (*β*-TCP), bioceramics, and hydroxyapatite (HA), have been used for bone regeneration. They can be manufactured at lesser costs compared to heterologous biomaterials; however, their resorption is not always ideal. Although inadequate resorption does not cause problems in bone grafting for mucosa-supported prosthesis, in preimplant grafting, the residual grafted material could affect the osseointegration.

### 3.3. Matrices

A matrix is defined as a microscopic structure inserted into the body to give form to a macroscopic and organized structure [28]. Matrices are divided as organic and inorganic matrices.

Organic matrices include collagen type I—the most abundant protein in human and animal tissues, which is required for the structural maintenance of tissues. Collagen has also been included in the group of polymeric materials in a subsequent section, where it will be discussed in detail.

All inorganic matrices have common features, such as fragility, no susceptibility to corrosion, and small fatigue resistance. The most significant property of inorganic matrices is their long resorption time in the body associated with the absence of any induced inflammatory response. These materials have been used in dentistry since the 1980s; however, none of them possesses the properties of osteogenesis and bone induction [17]. HA and calcium phosphate (CaP) are the commonly used inorganic matrices. CaP-based inorganic matrices can be further classified as ceramic and cement matrices. Ceramic matrices are divided into bioglass (BG) and HA ceramics, whereas *β*-TCP is included in a subgroup of cement matrices [17].

HA can be derived from animal bone, coral, or be manufactured as purely synthetic HA. HA has several disadvantages that include reduced mechanical resistance, long resorption time, and difficulty in controlling pore size [24]. The *β*-TCP-based scaffolds are preferred to HA-based scaffolds as they are easier to manufacture and can be shaped with suitable morphologies and controlled pore sizes. Moreover, the resorption rate of *β*-TCP-based scaffolds is 3–12-fold faster than that of HA-based ones, but it is also equally difficult to precisely predict the time taken for their resorption. *β*-TCP-based scaffolds have low mechanical strength, which further decreases with the increase in pore size and texture. For these reasons, CaP cannot be used alone as a scaffold in conditions of immediate loading [17].

The BG ceramics are biocompatible glass (approved by the FDA) and entirely synthetic [29]. BG is composed of oxides of silicon, sodium, calcium, phosphorus, and boron, although the final chemical composition is extremely variable according to the percentage of the various elements present. For this reason, the final characteristics of the product will differ in BGs, and it may be very difficult to clearly obtain the final product properties, even after lengthy industrial and chemical studies. It has also been shown that the high percentage of ions released by the insertion of BG in the body induces intra- and extracellular responses by stimulating osteoblast differentiation and revascularization [30, 31, 33]. Moreover, BGs demonstrate controlled resorption in optimal time, efficient bioactivity, and the ability to modulate cell migration and can form chemical bonds with the tissues with which they come in contact. If placed in contact with a liquid medium, they form a layer of a hydroxycarbonate gel of calcium and silica, which facilitates the absorption of proteins used by osteoblasts to produce matrix [32]. Furthermore, BGs have an advantageous peculiarity in their cortical bone-like modulus of elasticity [24], which can support the revascularization, enzyme activity, adhesion, growth, and differentiation of osteoblasts [17, 26]. Despite these exceptional characteristics, this biomaterial has low resistance to load, similar to other inorganic matrices [24, 32].

### 3.4. Polymeric Materials

Polymeric materials are classified as natural and synthetic polymers. Natural polymers are organic in origin and the most representative organic polymer is collagen. Collagen is used in bone and periodontal regeneration, often in combination with other grafting materials such as HA and *β*-TCP. Other organic polymers used as scaffolds (but less frequently for bone regeneration) include alginate, hyaluronic acid, chitosan, and peptide hydrogel [17]. Although these materials are biocompatible, they have a major disadvantage in being water soluble. For this reason, their use in bone regeneration is limited and they can be used only in combination with other materials, such as HA and TCP [17].

Synthetic polymers are manufactured industrially from inorganic sources. They are classified as absorbable and nonabsorbable polymers. The resorbable polyesters are predominant among synthetic polymers. They include polylactic acid (PLA), polyglycolic acid (PGA), polylactic-polyglycolic acid (PLGA), polyethylene glycol (PEG), PEG with PLGA (PEG-PLGA), and polycaprolactone (PCL) [25].

The most nonabsorbable polymer used in bone regeneration is polytetrafluoroethylene-expanded (e-PTFE). This polymer is used in the form of a membrane to cover bone grafts and it is used as a barrier between the graft material and the soft tissues of the flap to inhibit the early onset of gingiva-derived fibroblast formation, which could provoke scar tissue formation, before the proliferation of osteoblasts. e-PTFE is commonly anchored with metallic or synthetic absorbable pins and subsequently removed before the implant placement [32].

Collagen is a part of the bone matrix before primary ossification. It is biocompatible and biodegradable and has poor mechanical properties. Since it is already present in the body, it does not induce toxicity-based inflammatory or immunological responses when grafted [34]. It can be easily manipulated for the formation of 3D scaffolds with controlled porosity. The increase in scaffold porosity diminishes its mechanical characteristics [34]. The cytocompatibility of this polymer also makes it an excellent substrate for the proliferation of MSCs in vitro [35]. It is a hydrophilic material, whose permeability is essential for cell migration. The term permeability refers to the capacity of fluids to pass through tissues or membranes, and thus, the higher the scaffold compression, the greater the reduction in its permeability. Consequently, collagen is not suitable as a scaffold that can tolerate excessive loads, because as it gets squeezed or compressed, the quality of the collagen scaffold gets reduced [36]. Owing to its malleability, collagen is often combined with other materials (PLA, PGA, BG, and HA) in order to improve its mechanical characteristics, thus forming a complex or composite scaffold. This material can also promote engraftment and cell differentiation [34]. Collagen scaffolds have also been used to efficiently vehicle growth factors, but this field of research is still under development [17, 35].

PLA and PGA are synthetic polymers with excellent biomaterial characteristics that are dependent on the ability to control their synthesis, which influences the final surface characteristics [37]. Quantitatively, there are no limitations to their production and they are degraded in the body by chemicals and not cell-mediated processes [38]. The PLA and PGA polymers are cleaved into their respective monomers (lactic acid and glycolic acid, resp.) that are eliminated through different metabolic pathways. Their rapid degradation is also a disadvantage as it could cause early failure of the graft. Additionally, intracellular degradation of an acid can induce an inflammatory response [37, 39]. To reduce inflammation, hybrid scaffolds have been created, combining PLA and PGA with BGs and CaP [38, 40]. These polymers also have other disadvantages, such as low mechanical strength, difficulties associated with their production, and their uncertain interaction with cells. PGA degrades more rapidly than PLA. However, both are degraded too quickly for bone regeneration. Due to this reason, they are never used individually, but only as a combination in the form of PLGA 12 : 13 [24].

The polylactic-polyglycolic acid (PLGA) is a copolymer obtained by the union of lactic and glycolic acid through ester bonds. The composition of the final polymer chains will influence the degradation time, prolonging the half-life of the composite polymer in the oral cavity once applied in situ [35]. The different relationships between the two monomers and the different sequences that can be obtained greatly increase the variability of the final scaffold used in clinical practice, with several different formulations and resorption times. The 50 : 50 combination of PLA and PGA is less resistant, while the presence of the right- and left-handed monomers increases the resistance of the biomaterial [24]. The relationship between hydrophilicity and hydrophobicity and the balance of the crystalline structure, in turn, increases resorption times. It is also possible to control the morphology and diameter of the pores, as well as all other surface features [37, 39]. Currently, this polymeric bone substitute is extensively used for bone regeneration in dentistry and it has been combined with growth factors and MSCs to obtain good results [37]. Moreover, it can be fabricated in different forms: hydrogels, microspheres, blocks, and fibers [37, 39].

PEG is a polyether with a high molecular weight and is very resistant to resorption. It has been used in combination with MSCs and peptides with good results; in addition, it has been used as a scaffold for neuronal regeneration in the treatment of pathologies of the central nervous system [35].

PCL is an aliphatic polyester that is lesser known among the synthetic polymers. It has good mechanical characteristics and very long resorption times (of up to three years) and degrades via hydrolysis of the ester bonds [24]. It has been combined with HA and chitosan to form hybrid scaffolds with better mechanical resistance and has also been used in association with MSCs and growth factors [24, 41, 42].

### 3.5. Composite Scaffolds

The scaffolds described in the preceding sections are the most commonly used scaffolds in bone regeneration in dentistry. In some cases, it is possible to combine some of these materials to improve their mechanical characteristics and osteoconductivity. In fact, the wide range of biomaterials can be further widened if we consider combined (composite) scaffolds, obtained by the union of several components. Composite scaffolds obtained when PLA is enriched with dicalcium phosphate [38] or PGA and PLGA are combined with HA or *β*-TCP and can increase the degradation time and improve the mechanical properties of the scaffolds [36, 43, 44]. Scaffolds containing HA reinforced with collagen have been developed to overcome the mechanical strength limitations of collagen and stimulate the differentiation of stromal cells in vitro and in vivo [45]. Collagen was also enriched with growth factors to induce osteogenesis or associated with MSCs and polypeptides in order to improve cellular colonization [35, 46, 47]. Furthermore, other novel combined scaffolds have also been developed, such as PCL and bioactive glass coated with magnesium to implement bioactivity [48]. Metallic magnesium was also used in association with PLGA in order to stimulate in vitro stromal cells proliferation [71]. Even though the combination of different materials is usually convenient, it is essential that the design of these combined scaffolds must be accurate in order to optimize the results, minimize the disadvantages of each material, and enhance the advantageous properties.

Scaffolds enriched with HA have had the best outcomes in maxillofacial surgery [43]. In particular, different collagen formulations enriched with nano-HA have been created to increase migration and differentiation of progenitor cells involved in bone regeneration [45, 72]. These scaffolds were also tested in vitro with MSCs with the aim to graft bioactive scaffolds enriched with them [71]. PLGA scaffolds have been enriched with HA to slow the graft resorption time and enhance the mechanical properties; surprisingly, this combination also increased cell engraftment and the amount of newly formed bone tissue [43, 44].

## 4. Scaffolds with Stem Cells

Several scaffolds currently used in dental tissue engineering have been discussed in this review. Each biomaterial has a specific chemistry, composition and structure, and degradation profile and offers the possibility for modification. The combination of these scaffolds with stem cells represents the gold standard for future clinical treatments in dentistry-based bone regeneration approaches [1]. Different research groups and researchers have extensively described the positive association between scaffold and stem cells for bone regeneration. For this reason, the in-depth understanding of the molecular interactions between different scaffolds, stem cells, and their in situ microenvironment remains the main objective that needs to be achieved in regenerative medicine.

Among the organic scaffolds, collagen, despite its poor mechanical properties, is one of the most investigated materials. Its cytocompatibility and hydrophilicity make it perfect for cell adhesion in short time periods and, as an organic matrix, it effectively promotes cell viability, proliferation, and adhesion to the scaffold material [15]. The association between collagenous scaffold and stem cells has been extensively studied in vitro by evaluating the specific cell behavior, as well as in vivo both in animal studies and in human clinical trials. In particular, Kawase et al. demonstrated that the periosteum sheets coated with collagen enhanced initial adhesion of periosteum segments, improved cell growth, and increased the efficiency of implantation in periodontal therapy [47]. Moreover, the combination of poly-dL-lactic acid (PDLLA) and collagen promoted cell proliferation and osteogenic differentiation of MSCs as compared to MSCs seeded on the simple PDLLA/gelatine scaffolds [46, 47]. Zhang et al. showed that newly formed tissue, regenerated by DPSCs seeded on collagen sponges in vivo, appeared to be similar to connective tissues rather than dentin-like tissues [32, 73]. For this reason, collagen sponges are frequently used in combination with other materials, such as HA or PLGA, in order to enhance the mechanical properties of the scaffold and to overcome the inadequate production of mineral matrix deposition [31–33]. However, further studies will be necessary in this field before human clinical trials can be conducted.

Inorganic matrices have been studied in vivo and in vitro. HA is a commonly used scaffold in regenerative oral surgery in combination with stem cells. Several studies have shown that a scaffold composed of porous HA and MSCs with controlled and interconnected porosity facilitate bone regeneration, promoting the deposition of more bone matrix than simple HA [4, 41, 72]. However, poor cell adhesion and fast resorption remained the major problems associated with this biomaterial.

Calcium phosphate, dicalcium phosphate, and TCP are inorganic matrices widely used as scaffolds for their excellent mechanical and physical properties [27]. Their slow resorption and the ability to form chemical bonds with tissues make them suitable for bone regeneration. Gandhimathi et al. demonstrated that the physicochemical characteristics of these matrices promote cell communication, which results in matrix production [74]. In contrast, other studies [28, 74–77] have shown that, owing to the strong hydrophobicity of this material, it does not promote cell adhesion. Surface treatments or association with biomolecules, such as collagen, alginate, or polypeptides, are therefore necessary to facilitate the interaction between the cells and the biomaterial [75]. In fact, Wang et al. demonstrated that the incorporation of MSCs in nanoparticles mixed with calcium phosphate cements (CPC), inserted into cranial defects in nude rats, promoted bone regeneration [28]. Thus, these scaffolds are suitable for bone regeneration, showing good results in newly formed bone volumes in animal studies [75, 78].

Among many polymeric materials that have been widely studied and used in dentistry, PLGA has shown biocompatibility and good physical properties with suitable resorption times. For this reason, it is extensively used in dental procedure for bone healing [4]. Similar to calcium-based scaffolds, PLGA shows high hydrophobicity that entails the modification of PLGA surface in order to improve cell adhesion [79–81]. In fact, Chuenjitkuntaworn et al. showed that a novel fabricated 3D-PCL/HA scaffold possessed a good biocompatibility for osteoblasts, supporting cell growth and calcium deposition of three kinds of mesenchymal stem cells (DPSCs, BMSCs, and SHED) [79]. Other studies have suggested the potential roles of combined scaffolds of PLGA and PCL and organic matrices of collagen and MSCs in bone restoration by the creation of functional synthetic substitutes [45, 62, 81]. Graziano et al. demonstrated that the concave surface of PLGA 85 : 15, HA chips, and titanium increase cellular activity, matrix deposition, and the expression of bone-specific genes of human DPSCs in vitro [81]. Ryan et al. demonstrated that PLGA/PCL and HA/TCP scaffolds promoted in vitro cartilage matrices, whereas the in vivo application of these scaffolds supported progressive lamellar-like bone formation with mature bone marrow development for up to 8 weeks in mice [82].

We reviewed the most widely used biomaterials for scaffolds in oral implantology and oral surgery that are detailed in Table 1. Thus far, the literature suggests that it has not been possible to develop the “perfect” scaffold for maxillary bone regeneration. However, we can infer that the combination of different biomaterials represents the goal for future tissue engineering studies and clinical trials in dentistry.

## 5. Conclusion

Currently, dentists can select from among several biomaterials with different characteristics for bone regenerative surgery. Recent studies have shown that the use of combined scaffolds supplemented with mesenchymal stem cells is a safe procedure with predictable successful outcomes. In fact, several studies have demonstrated a good interaction between organic or inorganic scaffolds and adult stem cells in vitro. Thus, tissue engineering approaches have significantly and successfully enhanced the potential for bone regeneration in in vivo grafts. In the future, we will be able to develop custom-made 3D composite scaffolds that can be grafted with stem cells and precisely tailored to complement the exact shape of the bone defect. Therefore, future studies are needed to optimize approaches to facilitate complete restoration of defects in both hard and soft tissues.

## Figures and Tables

**Table 1:** Properties of scaffolds designed for dentistry tissue engineering.

Scaffolds	Advantages	Disadvantages	Preclinical and clinical studies
Bone graft	(i) Autologous bone is the gold standard (ii) A graft of cortical bone guarantees a good structural support and a reduced resorption (iii) A cancellous bone graft ensures an early revascularization resulting in rapid engraftment, less infection risk, and a shorter time for implant placement (iv) The allograft has no rejection or diseases transmission's risk (v) The xenografts costs are reduced compared with heterologous	(i) Newly formed tissue is slow and difficult (ii) The allograft has long and expensive process of sterilization and decellularization (iii) The xenografts do not have the best resorption time	(i) Autologous bone graft [18] (ii) Allografts [19, 20] (iii) Xenografts [21, 22]

Inorganic matrices			

(1) Hydroxyapatite (HA)	(i) Long resorption time in the body (ii) Absence of inflammatory response	(i) Fragility (ii) No susceptibility to corrosion (iii) Difficulty of controlling the size of pores (iv) Reduced mechanical resistance (v) Long resorption time in the body	Inorganic matrix [23–25]

(2) *β*-Tricalcium-phosphate (*β*-TCP)	(i) They are more easily produced and shaped in contrast to HA (ii) Suitable morphology (iii) Controlled pore sizes (iv) Resorption is 3–12 times faster than the HA	(i) Fragility (ii) No susceptibility to corrosion (iii) Difficult to predict precisely how long resorption will take (iv) Low mechanical strength, which decreases with the augment of the pores size and texture	[25]

(3) Bioglasses (BG)	(i) Accepted by the FDA (ii) Totally synthetic (iii) The final chemical composition is extremely variable (iv) Intra- and extracellular responses by stimulating osteoblast differentiation and revascularization (v) Controlled resorption time (vi) Bioactivity: good interaction with osteoblasts (vii) Modulate cell migration (viii) Form chemical bonds with the nearby tissue (ix) Cortical bone-like modulus of elasticity	(i) Low load resistance (ii) Fragility	[23, 24, 26–32]

Polymeric materials			

(1) Collagen	(i) Natural polymer (ii) Biocompatible (iii) Biodegradable (iv) Does not induce toxicity or inflammatory or immunological response (v) Easy to manipulate (vi) Porosity (vii) Permeability (viii) Malleability (ix) Used to vehicle growth factors	(i) Water soluble (ii) Limited bone regeneration (iii) Low mechanical properties	[23, 31–33]

(2) Polytetrafluoroethylene-expanded (e-PTFE)	Nonabsorbable synthetic polymer	Anchored with metal or synthetic absorbable pins	[30]

(3) Polylactic acid (PLA)	(i) No limits to their production (ii) Absorbable synthetic polymer (iii) Degraded in the body with chemical non-cell-mediated processes	(i) Rapid degradation (ii) Degradation of an acid causes an inflammatory response (iii) Low mechanical strength (iv) Difficulty of production (v) Dubious interaction with cells	[34–36]

(4) Polyglycolic acid (PGA)	(i) No limits to their production (ii) Absorbable synthetic polymer (iii) Degraded in the body with chemical non-cell-mediated processes	(i) Rapid degradation (ii) Degradation of an acid causes an inflammatory response (iii) Low mechanical strength (iv) Difficulty of production (v) Dubious interaction with cells	[34–36]

(5) Polylactic-polyglycolic acid (PLGA)	(i) Absorbable synthetic polymer (ii) Copolymer of PLA and PGA (iii) Control the morphology, diameter of the pores, and all surface features (iv) Combined with growth factors and MSCs	(i) The different relationships between the two monomers and the different sequences obtainable increase the variability of the final scaffold (ii) Hydrophobicity	[24, 36, 37]

(6) Polyethylene glycol (PEG)	(i) Resistant to resorption (ii) Used in combination with MSCs and peptides	Not used frequently in dentistry	[32]

(7) Polycaprolactone (PCL)	(i) Good mechanical characteristics (ii) Long resorption times (up to three years) (iii) Degrades by hydrolysis of the ester bonds (iv) Used with stem cells and polypeptides	Less studied	[24, 38, 39]

(8) Composite scaffolds	(i) The combination improves mechanical characteristics and osteoconductivity (ii) Overcome strength limitations and stimulate the differentiation of stromal cells in vitro and in vivo (iii) Implement bioactivity (iv) Increase migration and differentiation of progenitor cells	The design of these combined scaffolds must be necessarily accurate	[32–34, 40–48]
